# Surgical Strategies for Preservation of Pulmonary Valve Function in a Radical Operation for Tetralogy of Fallot: A Systematic Review and Meta-Analysis

**DOI:** 10.3389/fcvm.2022.888258

**Published:** 2022-07-13

**Authors:** Kang Yi, Dan Wang, Jianguo Xu, Xin Zhang, Wenxin Wang, Jie Gao, Wei Wang, Tao You, Jinhui Tian

**Affiliations:** ^1^Evidence-Based Medicine Center, School of Basic Medical Sciences, Lanzhou University, Lanzhou, China; ^2^Department of Cardiovascular Surgery, Gansu Provincial Hospital, Lanzhou, China; ^3^Gansu International Scientific and Technological Cooperation Base of Diagnosis and Treatment of Congenital Heart Disease, Lanzhou, China; ^4^Department of Geriatric Medicine, Xiangya Hospital, Central South University, Changsha, China; ^5^The First School of Clinical Medicine, Gansu University of Chinese Medicine, Lanzhou, China; ^6^The First Clinical Medical College, Lanzhou University, Lanzhou, China; ^7^Key Laboratory of Evidence Based Medicine and Knowledge Translation of Gansu Province, Lanzhou University, Lanzhou, China

**Keywords:** congenital heart disease surgery, systematic review, Tetralogy of Fallot (ToF), meta-analysis, pulmonary valve function

## Abstract

**Objective:**

To evaluate the efficacy and safety of different surgical strategies to preserve pulmonary valve function. Surgical procedures evaluated include intraoperative balloon pulmonary valvuloplasty (IBPV), pulmonary valve reconstruction, and commissurotomy and pulmonary cusp augmentation (PCA) in patients who underwent a radical operation for Tetralogy of Fallot (ToF).

**Materials and Methods:**

The five databases searched in the current study included the Cochrane Library, PubMed, China National Knowledge Infrastructure, VIP, and WanFang data. A systematic search for control trials was performed in each database from the start date of each database until December 2021. The Newcastle-Ottawa Scale (NOS) was used to evaluate the quality of included studies.

**Results:**

A total of 15 retrospective studies with a total number of 1,396 participants were included in this study. In subgroup 1 (IBPV vs. TAP), patients undergoing IBPV had a less degree of regurgitation at 1–2 years after the surgery. The reintervention rate increased in the IBPV group at 5 years. In subgroup 2 (pulmonary valve reconstruction vs. TAP), the degree of regurgitation decreased in the pulmonary valve reconstruction group at 1 month after the surgery. In subgroup 3 (valve-sparing operation vs. TAP), the comparison demonstrated decreased rates for surgical mortality and reintervention at 5–10 years after the surgery.

**Conclusion:**

We proposed that pulmonary valve function in a radical operation for ToF was preserved. IBPV, pulmonary valve reconstruction, and commissurotomy and PCA can be performed during the surgical procedure based on the developmental status and anatomical characteristics of the right ventricular outflow tract (RVOT), pulmonary valve, and pulmonary artery.

**Systematic Review Registration:**

[https://www.crd.york.ac.uk/prospero/], identifier [CRD42022300987].

## Introduction

As one of the most prevalent forms of cyanotic congenital heart disease, and one of the heart defects successfully repaired by congenital heart surgeons (1), Tetralogy of Fallot (ToF) has an incidence rate of approximately 1 in 3,500 neonates and accounts for 7–10% of all congenital cardiac malformations (2). Ventricular septal defect, right ventricular outflow tract (RVOT) obstruction, aortic overriding, and right ventricular hypertrophy are the main pathological features (3). From a genetic perspective, the etiology of ToF is a combination of genetics and environment, with a familial recurrence risk of 3% (2). Without timely surgical treatment, only 10% of patients could survive beyond the age of 20, others die before adulthood due to the consequences of secondary myocardial hypertrophy and heart failure caused by chronic hypoxia (4). Primary repair in the 1st year of life is the most prevalent strategy, with definitive corrective surgery as the best approach (5). Menaissy et al. have proposed that early total correction of ToF during the first 60 days of life can be performed with low mortality and good intermediate-term survival (6). As the first report of palliative Blalock–Taussig shunt in 1945 and surgical correction in 1954 (7), various techniques have been developed for the surgical treatment of ToF. The key to decreasing surgical mortality and to improving postoperative outcomes of a radical operation for ToF is a proper relief of obstruction to the RVOT.

Current approaches for RVOT reconstruction in a radical operation for ToF include transannular patch (TAP), commissurotomy, pulmonary cusp augmentation (PCA), pulmonary valve reconstruction, and intraoperative balloon pulmonary valvuloplasty (IBPV) (8). The TAP procedure is used to repair the RVOT with an incision on the distal main pulmonary artery extending proximally to divide the pulmonary annulus and suturing a patch for the reconstruction of RVOT. During commissurotomy, Hegar dilators are inserted serially at the pulmonary valve junction, aiming to be 1–2 mm higher than the normal pulmonary annular size calculated to repair RVOT (9). The PCA procedure creates a large anterior leaflet through a longitudinal incision from the pulmonic anterior leaflet to the annulus level, predicting and extending through the Hegar dilator, followed by the use of two patches to enlarge the anterior leaflet and to construct a new pulmonary sinus over the anterior leaflet (10, 11). In pulmonary valve reconstruction, a monocusp valve is used to reconstruct the pulmonary valve. During the IBPV procedure, a valvuloplasty balloon is introduced through the PV orifice inflated under direct vision, the size of the initial dilating balloon is determined by measuring the preoperative echocardiographic PV diameter measurement and the maximum intraoperative size of the rigid dilator (8, 12).

Transannular patch is recognized as a proven procedure that possesses the advantages of a high success rate and a certain effect. However, the predicted disadvantage of this procedure, the postoperative pulmonary regurgitation (PR), was affirmed in a significant number of patients who suffered pulmonary insufficiency (PI), heart failure, arrhythmia, and even sudden cardiac death (13, 14). Preserving or reconstructing pulmonary valve function is the best way to prevent PR and reduce the risk of adverse cardiovascular events (15). All procedures, including commissurotomy, PCA, pulmonary valve reconstruction, and IBPV can be performed to maintain pulmonary valve function to some extent. Commissurotomy exerts axial shear stress on the surrounding tissue, which may damage the valve and pulmonary annulus. It is a suitable procedure for patients with mild-to-moderate pulmonary stenosis accompanied by mild pulmonary valve dysplasia. PCA can make full use of the natural valve, which is suitable for patients with mild-to-moderate pulmonary valve dysplasia and pulmonary annular stenosis (16). Pulmonary valve reconstruction can effectively avoid postoperative PR, but the calcification of monocusp valve, thrombosis, endocarditis, and bad durability have restricted the application of monocusp in ToF (17). It is difficult to fully dilate the annulus using the IBPV procedure in patients with severe pulmonary valve dysplasia, and there is a risk of annulus tearing.

Based on the different indications and postoperative efficacy of ToF, we aimed to provide suggestions for the optimization of clinical treatments of ToF in the current systematic review, by aggregating studies and comparing IBPV, the valve-sparing operation (commissurotomy and PCA), and pulmonary valve reconstruction to TAP regarding surgical mortality, the incidence of PR, PI, and complications, the reintervention rate, cardiopulmonary bypass time, and aortic cross-clamp time.

## Materials and Methods

### Protocol and Registration

We registered a review protocol in the International prospective register of systematic reviews (PROSPERO)^1^ on 6 February 2022. The registration number is CRD42022300987 (Available at: https://www.crd.york.ac.uk/prospero/display_record.php?ID=CRD42022300987) (18).

### Search Strategy

A systematic literature search was performed in the Cochrane Library, PubMed, CNKI, VIP, and WanFang databases from the start date of each database through December 2021. Combinations of search terms include: “Tetralogy of Fallot,” “Fallot tetrad,” “valva trunci pulmonalis,” “pulmonary valve,” “pulmonary valve implantation,” “pulmonary cusp patch reconstruction,” “monocusp valve reconstruction,” “valvuloplasty,” “pulmonary valve restoration,” “extensive valvotomy,” “intraoperative balloon dilation,” “balloon pulmonary valvuloplasty,” “percutaneous balloon dilatation of the pulmonary valve,” “transannular patching,” and “transannular patch insertion.” Additional citations were supplemented by manually searching the references listed in all eligible studies.

### Study Screening

Two investigators independently identified articles that meet the following inclusion criteria: (1) Population: patients diagnosed with ToF. (2) Intervention and comparison: IBPV vs. TAP, pulmonary valve reconstruction vs. TAP, valve-sparing operation vs. TAP (commissurotomy or PCA vs. TAP). (3) Outcomes: surgical mortality, the incidence of PR, the incidence of PI, the incidence of complications, the reintervention rate, cardiopulmonary bypass time, and aortic cross-clamp time. (4) Study design: cohort studies. Studies without primary data and control group, are abstracts only, conference summary, meta-analysis, animal experiments, and duplicate data were excluded. In case of disagreement, a third investigator was included to decide whether the study was qualified. Agreement on the eligibility of the studies in question was achieved through a discussion among the three investigators.

### Data Extraction and Data Quality Assessment

Two investigators independently extracted data based on study design, title of the paper, name of the first author, publication year, follow-up time, patient characteristics, outcomes and common adverse events including PR, PI, death, tricuspid regurgitation, arrhythmia, low cardiac output syndrome, thrombus, etc. Conflicts about data extraction were discussed and resolved with a third investigator. The Newcastle-Ottawa Scale (NOS) was used by two investigators to assess the methodological quality of cohort studies (19).

### Statistical Analysis

Meta-analysis was performed among studies reporting outcomes of patients who underwent a radical operation for ToF. Data were divided into three subgroups for comparison with TAP. These three subgroups include IBPV vs. TAP, pulmonary valve reconstruction vs. TAP, and valve-sparing operation vs. TAP. The valve-sparing operation group includes both commissurotomy and PCA. In accordance with the Cochrane handbook for systematic reviews of interventions, we calculated the mean difference (MD) and the 95% confidence interval (CI) for continuous data and the risk ratio (RR) and the 95% CI for dichotomous data. Heterogeneity was tested using the Chi-squared test and the Higgins *I*^2^ test. The fixed-effects model was used if *p* > 0.1 or *I*^2^ < 50%, and the random-effects model was used otherwise. All of the analyses were performed with the software RevMan (Version 5.3).

## Results

### Search Results

**FIGURE 1:**
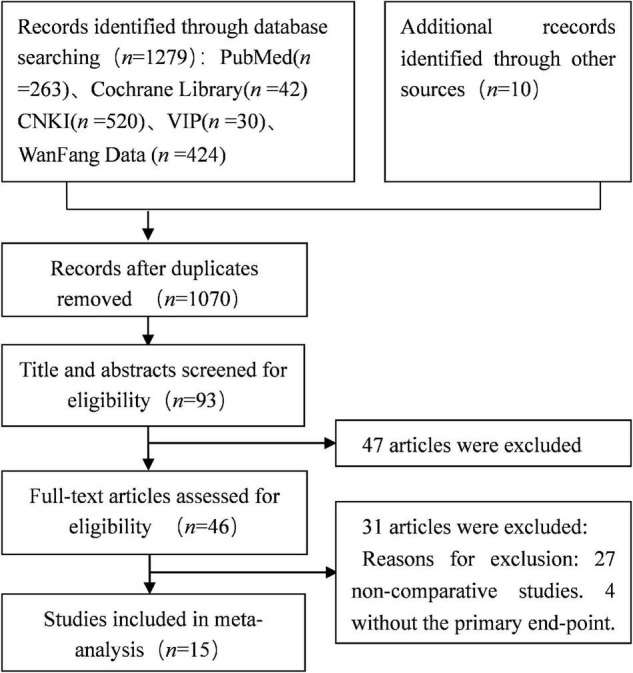
Flow diagram of study selection.

A total of 1,289 studies were obtained after the first round search following the search strategy described in section “Materials and Methods.” Studies of poor quality and repeated studies were excluded, and eventually 15 qualified retrospective studies with a total of 1,396 participants were included in this study (7, 8, 20–32). Figure 1 shows the literature screening strategy.

### Basic Characteristics of the Eligible Studies

The characteristics of the recruited studies include publication year, first author, country, study design, follow-up time, case source, comparison, and adjusted and/or unadjusted estimated effect with 95% CI are shown in Table 1.

**TABLE 1:** Characteristics of included studies in the meta-analysis.

Study	Region	Research time	Intervention	Total (*n*)	Dis.	Male/female	Age	Weight (Kg)	PVA-Z	McGoon index	Follow-up Rate	Outcomes
Zhou (20)	Henan, China	2012.01–2015.01	IBPV	36	17	10/7	28.29 ± 12.14 (m)	15.21 ± 2.99	−3.19 ± 0.53	1.53 ± 0.23	100%	①②④
			TAP		19	8/11	25.74 ± 9.75 (m)	13.76 ± 2.46	−3.32 ± 0.56	1.61 ± 0.35	94.74%	
Hofferberth et al. (21)	Boston, United States	2007.04–2015.12	IBPV	106	53	27/26	96 ± 55 (d)	/	−2.21 ± 0.55	/	100%	①④⑤
		1997.01–2006.12	TAP		53	36/17	83 ± 49 (d)	/	−2.26 ± 0.65	/	100%	
Robinson et al. (8)	Boston, United States	1997.01–2008.04	IBPV	143	32	/	94 ± 35 (d)	5.21 ± 0.89	−2.4 ± 0.96	/	86.96%	①②④⑤
			TAP		111	/	74 ± 45 (d)	4.56 ± 1.46	−2.79 ± 0.95	/	95.56%	
Zhang et al. (22)	Shanghai, China	2008.01–2013.01	PVR	87	19	/	/	/	/	1.69 ± 0.38	100%	①②④⑥⑦
			TAP		68	/	/	/	/	1.69 ± 0.36	100%	
Sasson et al. (23)	Tel Aviv, Israel	2003.01–2009.05	PVR	94	74	39/36	20.5 (0–196) (m)	9.35 (3.3–43)	−4 [−7–(−1)]	/	41.67%	①②④⑥⑦
			TAP		20	13/7	27 (4−192) (m)	9.35 (2.8−42.7)	−3.1 [−5–(−0.5)]	/	/	
Pande et al. (24)	Lucknow, India	2005.01−2007.12	PVR	40	16	/	7.5 (1.4−20) (y)	17 (6.0−38.0)	/	/	100%	①④
			TAP		24	/	11 (3.0−37.0) (y)	17.5 (7.0–55)	/	/	100%	
Turrentine (25)	United States	1990.06−1999.06	PVR	84	44	16/28	24.5 m (3 m–10 y)	/	/	/	90.91%	①
			TAP		40	17/23	33.1 m (7 m–10 y)	/	/	/	47.50%	
Chen et al. (26)	Zhengzhou, China	2005.01−2007.01	PVR	126	66	30/36	5.31 ± 1.64 (y)	17. 89 ± 3.86	/	1. 49 ± 0.05	/	④⑥⑦
			TAP		60	29/31	5.27 ± 3.64 (y)	18. 54 ± 5.46	/	1.47 ± 0.03	/	
Wang et al. (27)	Zhengzhou, China	2008−2012	PVR	46	20	/	/	/	/	/	95%	①②④
			TAP		26	/	/	/	/	/	96.15%	
Gan et al. (28)	Guangxi, China	2008.01−2008.12	PVR	61	31	18/13	/	/	/	/	/	①②④⑥⑦
			TAP		30	/	/	/	/	/	/	
Anagnostopoulos et al. (29)	San Francisco, United States	2001.11−2005.08	PCA	43	18	7/11	5.3 (1.3−9.1) (m)	6.4 (2.9−9.1)	/	1.47 (1.1−2.1)	94.44%	①④⑦
			TAP		25	12/13	3.2 (1.5−62.5) (m)	5.2 (3.3−14.3)	/	1.69 (1−2.3)	/	
Attanawanich et al. (30)	Bangkok, Thailand	1990−2004	PCA	93	55	/	48.33 ± 24.17 (m)	15.12 ± 4.38	/	2.16 ± 0.37	100%	①④⑤⑥⑦
			TAP		38	/	65.05 ± 30.64 (m)	16.22 ± 6.87	/	2.12 ± 0.41	100%	
Sen et al. (31)	NY, United States	2010.01−2014.05	PCA	51	19	/	156 (6–350) (d)	6 (1.9–8.5)	−2.51 (−5.80, −0.49)	/	100%	①④⑤
			TAP		32	/	109 (5–329) (d)	5.6 (2.3–10.5)	−3.07 (−9.00, −0.80)	/	/	
Xie et al. (32)	Jiangxi, China	2013.01−2016.06	Com.	272	35	24/11	7. 2 ± 9.9 (m)	5.5 ± 4. 3	−2.7 ± 1.5	1. 5 ± 0.3	/	①④⑥⑦
			TAP		237	125/112	8. 4 ± 1.6 (m)	5.1 ± 3.8	−2.9 ± 1.8	1. 4 ± 0.2	/	
Kim et al. (7)	Seoul, South Korea	1989.01−2005.12	Com.	114	57	38/19	9.3 ± 1.9 (m)	8.2 ± 1.3	−2.3 ± 1.3	2.4 ± 0.5	100%	①④⑤⑥⑦
			TAP		57	36/21	9.3 ± 2.2 (m)	8.3 ± 1.3	−2.1 ± 1.3	2.3 ± 0.4	100%	

*PVA-Z, Z-score of the pulmonary valve annulus diameter; PVR, pulmonary valve reconstruction; Dis., distribution; Com., commissurotomy.*

*①, surgical mortality;②, incidence of pulmonary regurgitation; ③, incidence of pulmonary insufficiency; ④, incidence of complications; ⑤, reintervention rate;⑥, Cardiopulmonary bypass time;⑦, aortic cross-clamp time.*

### Risk of Bias in Individual Trials

**FIGURE 2:**
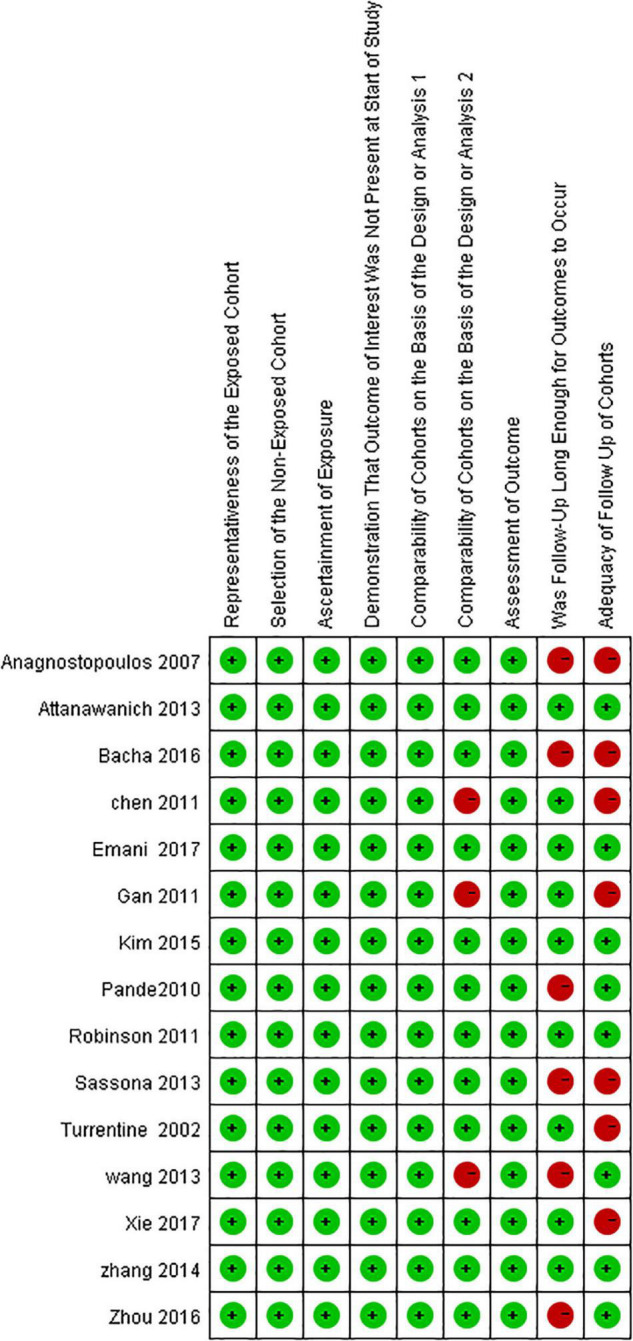
Assessment of the risk of bias for each included study.

The risks of bias in each included study were assessed following the NOS (Figure 2).

### Analysis

As shown in Table 2, three studies including 255 patients reported mortality in the subgroup of IBPV vs. TAP. No statistically significant difference was found between the IBPV and TAP groups [RR: 1.26, 95% CI (0.05, 30.06), *p* = 0.88] in terms of mortality rate, as shown in Supplementary Figure 1. Two studies including 141 patients reported the incidence of PR. The incidence of PR at 1–2 years of follow-up was significantly lower in the IBPV group than in the TAP group [RR: 0.48, 95% CI (0.33, 0.69), *p* < 0.0001] without significant heterogeneity (*p* = 0.37; *I*^2^ = 0%). The results are shown in Supplementary Figure 4. Two studies including 219 patients reported the reintervention rate. As shown in Supplementary Figure 6, the rate was statistically significant higher in the IBPV group than in the TAP group [RR: 12.43, 95% CI (3.36, 46.07), *p* = 0.0002] without significant heterogeneity (*p* = 0.93; *I*^2^ = 0%). Analysis of the data from three studies including 255 patients showed that no statistically significant difference was found between the IBPV and TAP groups [RR: 0.79, 95% CI (0.44, 1.40), *p* = 0.42] with significant heterogeneity (*p* < 0.00001; *I*^2^ = 93%). The results are shown in Supplementary Figure 8.

**TABLE 2:** Summary of forest plot in the meta-analysis.

Outcome	Surgery	Included studies	*N*	Effect size	Heterogeneity	Supplementary Figures
Mortality	IBPV vs. TAP	3	255	RR: 1.26, 95% CI (0.05, 30.06), *p* = 0.88	/	Supplementary Figure 1
	PVR vs. TAP	6	412	RR: 0.63, 95% CI (0.14, 2.75), *p* = 0.54	*p* = 0.92; *I*^2^ = 0%	Supplementary Figure 2
	VSO vs. TAP	5	573	RR: 0.18, 95% CI (0.04, 0.82) *p* = 0.03	*p* = 0.54; *I*^2^ = 0%	Supplementary Figure 3
PR	IBPV vs. TAP	2	141	RR: 0.48, 95% CI (0.33, 0.69), *p* < 0.0001	*p* = 0.37; *I*^2^ = 0%	Supplementary Figure 4
	PVR vs. TAP	4	288	RR: 0.28, 95% CI (0.11, 0.72), *p* = 0.008	*p* < 0.0001; *I*^2^ = 88%	Supplementary Figure 5
RR	IBPV vs. TAP	2	219	RR: 12.43, 95% CI (3.36, 46.07), *p* = 0.0002	*p* = 0.93; *I*^2^ = 0%	Supplementary Figure 6
	VSO vs. TAP	3	258	RR: 0.36, 95% CI (0.17, 0.77), *p* = 0.008	*p* = 0.74; *I*^2^ = 0%	Supplementary Figure 7
Complications	IBPV vs. TAP	3	255	RR: 0.79, 95% CI (0.44, 1.40) *p* = 0.42	*p* < 0.00001; *I*^2^ = 93%	Supplementary Figure 8
	PVR vs. TAP	6	454	RR: 0.55, 95% CI (0.17, 1.78) *p* = 0.32	*p* < 0.00001; *I*^2^ = 98%	Supplementary Figure 9
	VSO vs. TAP	5	573	RR: 0.90, 95% CI (0.65, 1.25) *p* = 0.54	*p* < 0.00001; *I*^2^ = 96%	Supplementary Figure 10
CBT	PVR vs. TAP	4	368	MD = 5.51, 95% CI (−8.26, 19.29) *p* = 0.43	*p* < 0.00001; *I*^2^ = 92%	Supplementary Figure 11
	VSO vs. TAP	3	479	MD = 13.14 95% CI (−10.99, 37.28) *p* = 0.29	*p* < 0.00001; *I*^2^ = 93%	Supplementary Figure 12
ACT	PVR vs. TAP	4	368	MD = 8.36, 95% CI (−5.00, 21.71) *p* = 0.22	*p* < 0.00001; *I*^2^ = 95%	Supplementary Figure 13
	VSO vs. TAP	4	522	MD = 11.89, 95% CI (−9.70, 33.48) *p* = 0.28	*p* < 0.00001; *I*^2^ = 96%	Supplementary Figure 14

*PR, pulmonary regurgitation; RR, reintervention rate; CBT, cardiopulmonary bypass time; ACT, aortic cross-clamp time; TAP, transannular patch; IBPV, intraoperative balloon pulmonary valvuloplasty; PVR, pulmonary valve reconstruction; VSO, valve-sparing operation.*

Analysis on the same factors was performed to compare the pulmonary valve reconstruction group and the TAP group. As demonstrated in Supplementary Figures 2, 5, 9, 11, 13, the pulmonary valve reconstruction group and the TAP group were compared with respect to the surgical mortality rate, PR in a 1-month follow-up, the incidence of complications, cardiopulmonary bypass time, and aortic cross-clamp time, respectively. The detailed data were listed below in the order of the factors listed above. Surgical mortality: No statistically significant difference between the two groups [RR: 0.63, 95% CI (0.14, 2.75), *p* = 0.54] with no significant heterogeneity of samples (*p* = 0.92; *I*^2^ = 0%). Six studies having 412 patients were included in the mortality rate comparison. PR in a 1-month follow-up: Four studies including 288 patients reported the incidence of PR. PR was significantly lower in the pulmonary valve reconstruction group [RR: 0.28, 95% CI (0.11, 0.72), *p* = 0.008] with significant heterogeneity (*p* < 0.0001; *I*^2^ = 88%). Incidence of complications: six studies including 454 patients reported the incidence of complications. No statistically significant difference was found between the two groups [RR: 0.55, 95% CI (0.17, 1.78), *p* = 0.32] with significant heterogeneity (*p* < 0.00001; *I*^2^ = 98%). Cardiopulmonary bypass time: four studies including 368 patients reported cardiopulmonary bypass time. No statistically significant difference was found between the two groups [MD = 5.51, 95% CI (–8.26, 19.29), *p* = 0.43] with significant heterogeneity (*p* < 0.00001; *I*^2^ = 92%). Aortic cross-clamp time: four studies including 368 patients reported aortic cross-clamp time. No statistically significant difference was found between the two groups [MD = 8.36, 95% CI (–5.00, 21.71), *p* = 0.22] with significant heterogeneity (*p* < 0.00001; *I*^2^ = 95%).

In the valve-sparing vs. TAP subgroup, similar comparisons were performed using the same methods. The results were listed as follows: Surgical mortality: five studies including 573 patients reported mortality. The mortality rate was significantly lower in the valve-sparing group [RR: 0.18, 95% CI (0.04, 0.82), *p* = 0.03] without significant heterogeneity (*p* = 0.54; *I*^2^ = 0%). Reintervention rate: three studies including 258 patients reported the reintervention rate. It was significantly lower in the valve-sparing group [RR: 0.36, 95% CI (0.17, 0.77), *p* = 0.008] with no significant heterogeneity (*p* = 0.74; *I*^2^ = 0%). Incidence of complications: five studies including 573 patients reported the incidence of complications. No statistically significant difference was found between the two groups [RR: 0.90, 95% CI (0.65, 1.25), *p* = 0.54] with significant heterogeneity (*p* < 0.00001; *I*^2^ = 96%). Cardiopulmonary bypass time: three studies including 479 patients reported cardiopulmonary bypass time. No statistically significant difference was found between the two groups [MD = 13.14, 95% CI (−10.99, 37.28), *p* = 0.29] with significant heterogeneity (*p* < 0.00001; *I*^2^ = 93%). Aortic cross-clamp time: four studies including 522 patients reported aortic cross-clamp time. No statistically significant differences were found between the two groups [MD = 11.89, 95% CI (–9.70, 33.48), *p* = 0.28], with significant heterogeneity (*p* < 0.00001; *I*^2^ = 96%). All data were shown in Table 2 and online Supplementary Figures 3, 7, 10, 12, 14.

## Discussion

Tetralogy of Fallot is one of the most prevalent cyanotic congenital heart diseases, presenting a poor prognosis without surgical treatment (4). Definitive corrective surgery is the best option for patients with ToF. Along with the elongation of the follow-up term, increased comprehensiveness of data collected during follow-up, and the sensitivity of the examinations, complications after a radical operation for ToF have drawn more attention (33). TAP has been shown to be an approach that possess the advantages of some effect to relieve pulmonary annular stenosis, but there are disadvantages about TAP including poor long-term postoperative efficacy and poor survival rate caused by obvious postoperative PR (34). Recently, efforts have been focused on surgical strategies to preserve pulmonary valve function with the aim of reducing postoperative regurgitation and improving the quality of long-term survival (16). Our meta-analysis compared the three different types of approaches, IBPV, pulmonary valve reconstruction, and valve-sparing operation, with TAP on various parameters, attempting to provide suggestions for the optimization of clinical treatment for ToF.

Comparison between the IBPV group and the TAP group in a radical operation for ToF revealed that the degree of regurgitation were lower in the IBPV group at 1–2 years after the surgery [RR: 0.48, 95% CI (0.33, 0.69), *p* < 0.0001], the reintervention rate increased in the IBPV group at 5 years [RR: 12.43, 95% CI (3.36, 46.07), *p* = 0.0002]. There was no difference in terms of surgical mortality and the incidence of complications.

Intraoperative balloon pulmonary valvuloplasty in infants with ToF is a relatively safe palliative surgery and appears to produce adequate palliation in most patients. It allows the growth and increased pulmonary blood flow (35, 36). Continuous improvements in surgical techniques and postoperative management have made a radical operation for ToF to achieve a good outcome in early infancy with a low risk. IBPV, as an effective strategy to preserve the integrity of the PV annulus, has been gradually applied in a radical operation (12). A valvuloplasty balloon was introduced through the PV orifice and inflated under direct vision, presenting a significant longitudinal PV growth after the surgery and the normalization of annular size over time (8). In our study, most patients in the TAP group presented severe PR immediately after the surgery, while the degree of regurgitation and the incidence of severe regurgitation in the IBPV group were significantly decreased at 1–2 years after the surgery. However, the Boston group reported that patients exhibited progressive PR with IBVP at an midterm follow-up and that the absence of at least moderate PR at 5 years was only 43% (21). Long-term efficacy of the valve-sparing repair with IBVP warrants further prospective analyses. There is a higher postoperative reintervention rate at 5 years after the surgery with IBPV in this study, which may be related to residual obstruction from inadequate dilatation in patients with severe pulmonary valve dysplasia, or to annulus tearing in overdilatation. As for the residual pulmonary valve stenosis (PS) resulting from minimal RVOT enlargement strategies, it has been suggested that a proper relief of RVOT obstruction with acceptable residual stenosis is more advantageous than aggressive RVOT enlargement in the long-term outcome of these patients with ToF repair, which was reported in the study of Yoo et al. This could make the right ventricle (RV) diastolic properly to protect against RV dilatation caused by PR (37). Therefore, in a radical operation for ToF, we recommend IBPV for patients with mild-to-moderate PS or pulmonary annulus stenosis.

By comparing the pulmonary valve reconstruction group to the TAP group in a radical operation for ToF, it was found that the degree of regurgitation decreased in the pulmonary valve reconstruction group at 1 month after the surgery. There were no significant differences in terms of surgical mortality, the incidence of complications, cardiopulmonary bypass time, and aortic cross-clamp time. Pulmonary valve reconstruction effectively alleviates regurgitation caused by the mismatch between the enlarged annulus and the original valve in TAP. Meanwhile, the operation time is not significantly increased. From a safety perspective, the risks of death and complications are not increased either.

Durability and tolerance of the reconstructed valve are the key issues in pulmonary reconstruction. Calcification, stenosis, and a poor biological function of the reconstructed valve will cause adverse postoperative efficacy and survival rate. A variety of patch materials have been used to solve this problem in clinical practice, including autologous pericardial patches, bovine pericardial patches, polytetrafluoroethylene (PTFE) membranes, Gore-Tex, bovine jugular vein (BJV), and homografts (2, 38). There is still a lack of ideal materials for valve reconstruction. In the early stage, fresh autologous pericardium and pericardium plus a Dacron patch were used for repair, but postoperative regurgitation did not decrease significantly (39, 40). In our present study, four included studies reported the degree of PR. Three of the four used pericardial patches (21, 27, 28), and one used PTFE (23). By comparison, the lowest degree of PR was paired with PTFE. The average follow-up duration in the four studies was 2 years, lacking the long-term follow-up data. Durability and functionality of the pulmonary valve need to be observed over the long term. At present, more attention is being paid to PTFE and BJV. Some studies have shown that PTFE is relatively inexpensive and easy to construct, and remains free of significant stenosis and calcification or pulmonary embolization in the majority of patients (41, 42). Meanwhile, in the study by Sasikumar et al., patients who were using the monocusp valve suffered the loss of value function in a significant proportion and PR also progressed at 1 year. Although no conclusions cannot be reliably drawn regarding the predictors of adverse outcomes due to the limited sample size in this study, some uncertainty remains (17). Being a tissue, there is a natural continuity in the BJV between the valve and jugular vein patch that allows infundibular shaping without the need for additional materials. It is also relatively cheap and easy to construct (43). Comparing with homografts, the use of BJV was associated with a significantly higher incidence of bacterial endocarditis, leading to some unpredictable risks (44). The conflicting reports regarding the performance and durability of the reconstructed valve may be related to surgical techniques and materials used in valve reconstruction. Based on current studies, we recommend the use of PTFE or BJV in a radical operation for patients with ToF who have severe pulmonary valve dysplasia or valve stenosis to minimize postoperative complications and to improve long-term surgical efficacy.

Comparison of the valve-sparing operation group with the TAP group revealed that surgical mortality and the reintervention rate at 5–10 years after the surgery were lower in the valve-sparing operation group than in the TAP group. There was no significance regarding the incidence of complications, cardiopulmonary bypass time, and aortic cross-clamp time. The valve-sparing operation includes commissurotomy and PCA, preserving the shape and function of the natural valve. In the repair by commissurotomy, the valve root attached to the arterial wall is kept intact, and the effective areas of the PV orifice and the valve are increased. Due to the high requirements for the natural valve, commissurotomy is only suitable for patients with mild-to-moderate PS or mild pulmonary valve dysplasia, and there is a risk of intraoperative damage to the valve and the valve annulus, so its clinical application is limited (16). PCA uses patches to create a large anterior leaflet based on the natural valve, which has a wide range of clinical applications compared to commissurotomy. The preservation of the pulmonary valve coaptation mechanism, as well as the cusp suspension mechanism, keeps the natural valve configuration and function, so as to persist growth potential and reduce the incidence of PI, which is also an important element in reducing the reintervention rate (45). In our study, only two included studies reported the follow-up data of PI. Compared to TAP, the incidence of PI in the valve-sparing operation decreased (29, 30). Durability and tolerance of the augmentative valve are also the key issues in clinical practice. The most routinely applied is glutaraldehyde-treated autologous pericardium. In the study by Hua et al., fresh autologous pericardium was used instead of glutaraldehyde-treated pericardium. Pathological findings and physical characteristics, elasticity and softness, persisted well with fresh autologous pericardium, but long-term data were not enough to confirm completely (10). In our present meta-analysis, we recommend PCA for patients without severe natural pulmonary valve or pulmonary annular stenosis and dysplasia as well as the secondary operation after TAP that preserves the natural pulmonary valve. We recommend commissurotomy for patients with mild-to-moderate PS or mild pulmonary valve dysplasia.

Previous findings on the radical operation for ToF demonstrate that, for patients with severe pulmonary annular stenosis but with a well-developed pulmonary valve, TAP can relieve the obstruction with some effect, but did so at the expense of obvious PR after the surgery, which results in chronic right ventricular volume overload, inevitably leading to progressive right ventricular dilation- and dysfunction-associated reintervention. Surgical strategies for preserving pulmonary valve function can preserve completeness of the pulmonary valve to the extent as much as possible, relieving the obstruction effectively and reducing the incidence of pulmonary valve regurgitation, so as to reduce an adverse effect on the right ventricular function caused by long-term regurgitation. Compared with TAP, IBPV preserves the integrity and growth of the PV annulus. Pulmonary valve reconstruction alleviates the regurgitation caused by the mismatch between the enlarged annulus and the original valve in TAP. The valve-sparing operation preserves the natural valve configuration and function.

Our meta-analysis summarized currently available surgical strategies to preserve pulmonary valve function in a radical operation for ToF, including pulmonary valve reconstruction, IBPV, and valve-sparing operation. Valve-sparing operation includes commissurotomy and PCA. IBPV can be performed to reduce the degree of regurgitation, shorten the operation time for patients with mild-to-moderate PS or pulmonary annulus stenosis, achieving a significant improvement after the surgery. Pulmonary valve reconstruction can reconstruct functional valves to better reduce PR in patients with severe PS or pulmonary valve dysplasia. Valve-sparing operation preserves the natural valve configuration and function so as to reduce the reintervention rate and surgical mortality in patients without severe natural pulmonary valve or pulmonary annular stenosis and dysplasia. According to the development and anatomical characteristics of the RVOT, pulmonary valve and pulmonary artery, we recommend proper surgical strategies to preserve pulmonary valve function during a radical operation for ToF to achieve long-term postoperative efficacy.

Our study has certain limitations. First, the eligible studies are retrospective, which may affect the strength of resultant demonstration. Second, the unbalanced development of the technique in different medical centers may cause heterogeneity among the included studies. Third, the number of included studies in the IBPV subgroup is relatively small, which may affect the reliability of analytical results. Fourth, the lack of long-term follow-up in the pulmonary reconstruction subgroup makes the long-term efficacy of the specific technique unclear.

## Conclusion

In a radical operation for ToF, we recommend surgical strategies to preserve pulmonary valve function. According to the development and anatomical characteristics of the RVOT, pulmonary valve and pulmonary artery, IBPV, pulmonary valve reconstruction, and valve-sparing operation can be chosen.

## Data Availability Statement

The original contributions presented in the study are included in the article/Supplementary Material, further inquiries can be directed to the corresponding author/s.

## Author Contributions

KY, DW, TY, and JT: conceptualization. KY, DW, JX, XZ, WXW, and JG: data curation. KY, DW, JX, and XZ: formal analysis. TY: funding acquisition. KY, DW, TY, JX, XZ, WXW, JG, WW, and JT: investigation and writing – review and editing. KY, DW, JX, XZ, and JG: methodology. TY and JT: supervision. KY, DW, and TY: writing – original draft. All authors contributed to the article and approved the submitted version.

## Conflict of Interest

The authors declare that the research was conducted in the absence of any commercial or financial relationships that could be construed as a potential conflict of interest.

## Publisher’s Note

All claims expressed in this article are solely those of the authors and do not necessarily represent those of their affiliated organizations, or those of the publisher, the editors and the reviewers. Any product that may be evaluated in this article, or claim that may be made by its manufacturer, is not guaranteed or endorsed by the publisher.

## Footnotes

1
https://www.crd.york.ac.uk/PROSPERO
