# Supplementary material for: Surgical Strategies for Preservation of Pulmonary Valve Function in a Radical Operation for Tetralogy of Fallot: A Systematic Review and Meta-Analysis
Source: Front Cardiovasc Med. 2022 Jul 13;9:888258. doi: 10.3389/fcvm.2022.888258 (PMC9326161; doi:10.3389/fcvm.2022.888258)
Supplement: Supplementary file 1 [file Data_Sheet_1.docx]

**Supplementary figure legend**

Figure S1. Forest plot for surgical mortality in IBPV vs TAP.

Figure S2. Forest plot for surgical mortality in PVR vs TAP.

Figure S3. Forest plot for surgical mortality in VSO vs TAP.

Figure S4. Forest plot for incidence of pulmonary regurgitation in IBPV vs TAP.

Figure S5. Forest plot for incidence of pulmonary regurgitation in PVR vs TAP.

Figure S6. Forest plot for reintervention rate in IBPV vs TAP.

Figure S7. Forest plot for reintervention rate in VSO vs TAP.

Figure S8. Forest plot for incidence of complications in IBPV vs TAP.

Figure S9. Forest plot for incidence of complications in PVR vs TAP.

Figure S10. Forest plot for incidence of complications in VSO vs TAP.

Figure S11. Forest plot for cardiopulmonary bypass time in PVR vs TAP.

Figure S12. Forest plot for cardiopulmonary bypass time in VSO vs TAP.

Figure S13. Forest plot for aortic cross clamp time in PVR vs TAP.

Figure S14. Forest plot for aortic cross clamp time VSO vs TAP.


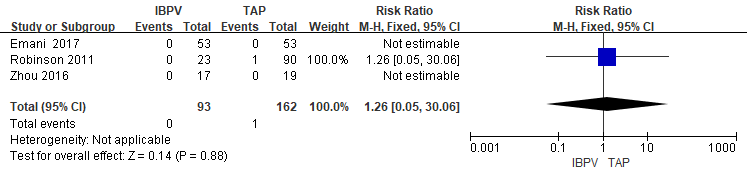


Figure S1. Forest plot for surgical mortality in IBPV vs TAP.


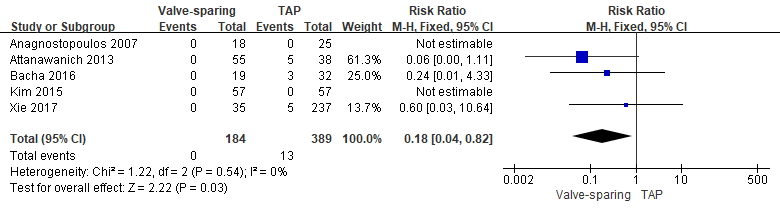


Figure S2. Forest plot for surgical mortality in PVR vs TAP.


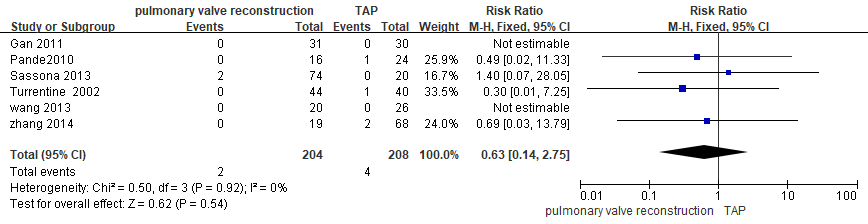


Figure S3. Forest plot for surgical mortality in VSO vs TAP.


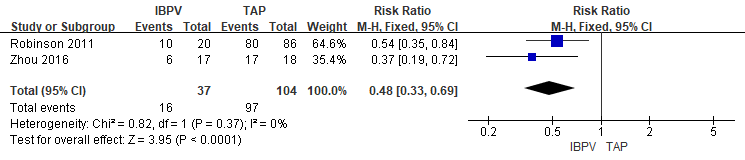


Figure S4. Forest plot for incidence of pulmonary regurgitation in IBPV vs TAP.


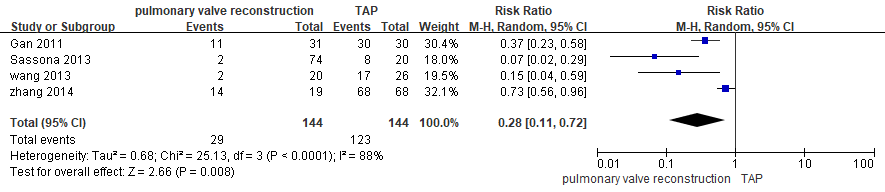


Figure S5. Forest plot for incidence of pulmonary regurgitation in PVR vs TAP.


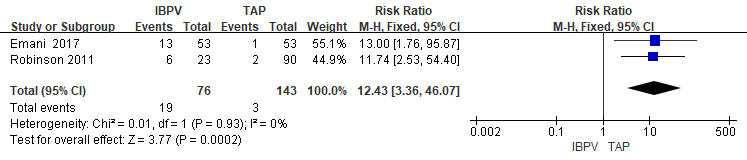


Figure S6. Forest plot for reintervention rate in IBPV vs TAP.


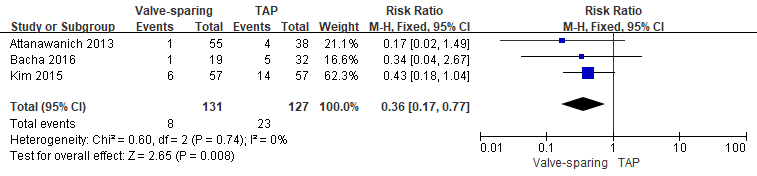


Figure S7. Forest plot for reintervention rate in VSO vs TAP.


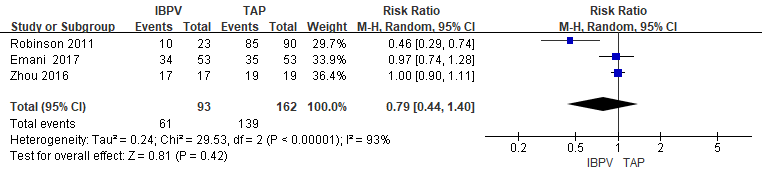


Figure S8. Forest plot for incidence of complications in IBPV vs TAP.


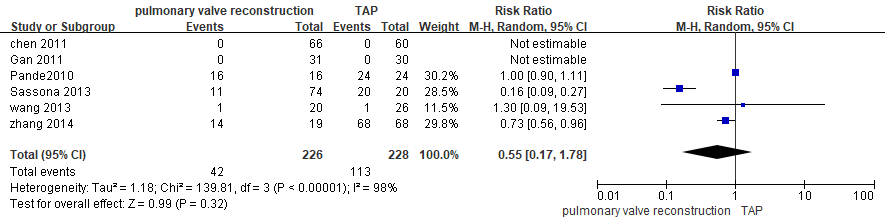


Figure S9. Forest plot for incidence of complications in PVR vs TAP.


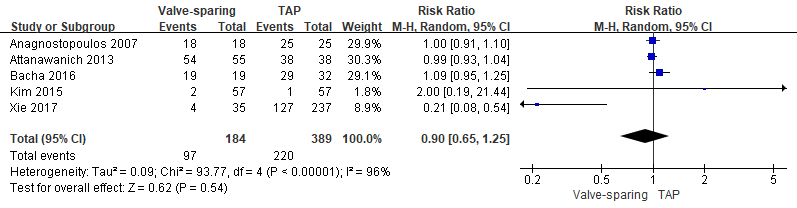


Figure S10. Forest plot for incidence of complications in VSO vs TAP.


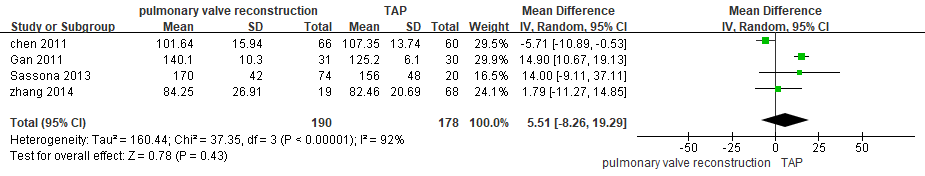


Figure S11. Forest plot for cardiopulmonary bypass time in PVR vs TAP.


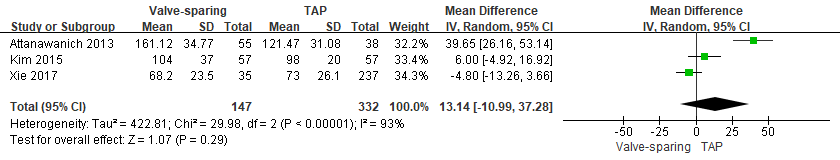


Figure S12. Forest plot for cardiopulmonary bypass time in VSO vs TAP.


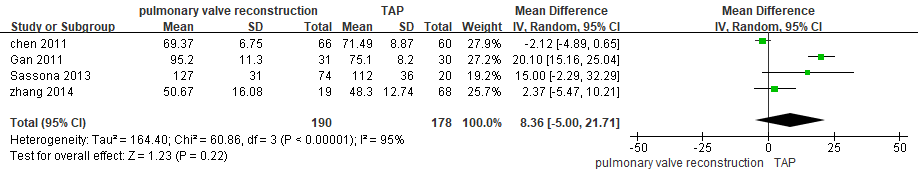


Figure S13. Forest plot for aortic cross clamp time in PVR vs TAP.


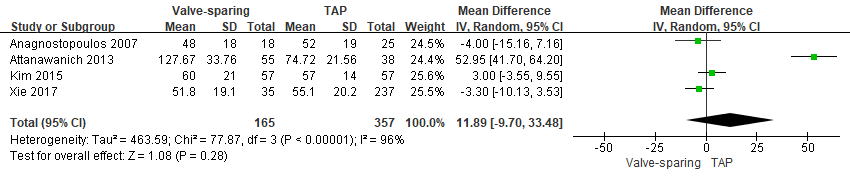


Figure S14. Forest plot for aortic cross clamp time VSO vs TAP.
